# AI-enhanced social media affordances and intercultural communication competence: engagement, identity integration, and English proficiency

**DOI:** 10.3389/fpsyg.2026.1960943

**Published:** 2026-09-14

**Authors:** Ruiqian Ma

**Affiliations:** School of Languages, Cultures and Societies, Faculty of Arts, Humanities and Cultures, University of Leeds, Leeds, United Kingdom

**Keywords:** AI-enhanced social media, cultural identity integration, English proficiency, intercultural communication competence, intercultural interaction engagement.

## Abstract

**Introduction:**

AI-enhanced social media increasingly provides university students with algorithmic recommendation, translation, writing assistance, pragmatic feedback, and cultural interpretation functions that can shape intercultural communication experiences. This study examined the associations between AI-enhanced social media affordances (AIESMA) and self-reported intercultural communication competence (ICC) among Chinese university students in business-related majors, focusing on the explanatory roles of intercultural interaction engagement (IIE) and cultural identity integration (CII), and the moderating role of self-reported English proficiency.

**Methods:**

A two-study design was employed using two independent samples totaling 1,200 Chinese university students in business-related majors. Study 1 involved 400 respondents and focused on instrument development and preliminary validation through expert review, cognitive interviews, exploratory factor analysis, and reliability assessment. Study 2 included 800 independent respondents and tested the measurement and structural models using partial least squares structural equation modeling, including direct, indirect, sequential indirect, moderation, and moderated mediation analyses.

**Results:**

AIESMA was positively associated with IIE and CII, whereas its direct association with self-reported ICC was not statistically significant after IIE and CII were included in the model. Both IIE and CII were positively associated with self-reported ICC. The indirect association between AIESMA and ICC through IIE was significant, whereas the indirect association through CII alone was not. The theoretically specified sequential indirect association through IIE and CII was significant. Self-reported English proficiency strengthened the AIESMA–IIE association and the conditional sequential indirect association, with stronger effects observed at higher proficiency levels.

**Discussion:**

The findings indicate that AI-enhanced social media affordances are associated with self-reported intercultural communication competence primarily through engagement-centered indirect associations rather than through a direct affordance-to-competence relationship. Cultural identity integration contributes to ICC within the theoretically specified engagement-to-identity pathway, while English proficiency functions as a modest boundary condition. These findings support the use of AI-enhanced social media as a guided environment for intercultural participation, reflection, identity negotiation, and professional communication practice in business education.

## 1 Introduction

Global business communication is increasingly organized through digital platforms where professional identities, consumer meanings, brand narratives, service evaluations, and intercultural relationships are negotiated. Recent social media marketing research shows that digital platforms have become central environments for consumer engagement, brand communication, and business interaction (Bartoloni et al., 2024). For Chinese business students, this transformation makes intercultural communication competence (ICC) an increasingly important professional capability because global business participation increasingly requires culturally appropriate interaction in English-mediated and digitally networked environments (Xu et al., 2025). ICC also refers to the ability to express oneself accurately, interpret cultural meanings, respond appropriately to people from other cultures, and participate effectively in intercultural communication (Canale and Swain, 1980). Competence is required for understanding different cultural values in business; thus, students need to learn about consumers’ expectations and brand stories around the world, as well as professional norms and how to build relationships in the digital era (Xu et al., 2025). Beyond English proficiency, ICC also includes cultural literacy, intercultural awareness, communication strategies, pragmatic appropriateness, multimodal cue interpretation, and interactional effectiveness (Zhou et al., 2024). At the same time, since most international business discussions on digital platforms are conducted in English or support English, students’ English proficiency may influence their access to these intercultural communication opportunities, their ability to understand English-mediated content, and their capacity to participate effectively in such interactions (Bachman and Palmer, 1996). Therefore, English proficiency should not be understood as equivalent to ICC, but as an important linguistic condition that may influence how students transform digital intercultural exposure into meaningful engagement.

Traditionally, ICC has been cultivated through classroom-based cultural instruction, foreign language courses, and structured intercultural exchange activities. Recent reviews of intercultural competence in higher education show that classroom-based, digitally mediated, and program-based approaches remain important, but their effectiveness depends on interaction, reflection, structured pedagogy, and contextually meaningful tasks rather than cultural knowledge transmission alone (Guillén-Yparrea and Ramírez-Montoya, 2023; Naranjo-Toro et al., 2026). These approaches remain valuable, but they may provide limited opportunities for continuous, authentic, and business-relevant intercultural participation. The rise of social media has changed this condition by making global business and consumer discourse more visible in students’ everyday digital lives (Bartoloni et al., 2024).

Artificial intelligence has further reshaped this platform ecology through algorithmic recommendation, machine translation, automatic subtitles, AI-assisted writing, AI-based explanation, content filtering, sentiment cues, and generative language support. Recent AI language education research shows that AI has been widely applied to writing support, automated feedback, reading, speaking, translation, and personalized learning (Huang et al., 2023; Kohnke et al., 2023). Generative AI research in higher education further suggests that AI tools can support learning autonomy, feedback, writing, and interaction, but also raise concerns about overreliance and shallow cognitive engagement (Kasneci et al., 2023; Qian, 2025). These functions can reduce barriers to accessing multilingual materials, support students’ comprehension of English comments and videos, assist in drafting or revising English responses, and explain unfamiliar cultural references. AI-enhanced social media (AISM) thus offers new affordances for intercultural contact, understanding, expression, feedback and cultural interpretation. However, affordance theory suggests that technological functions should not be regarded as automatic learning results, as affordances arise from the interplay among users, technology, and situated practice (Leonardi, 2011).

Although generative AI can produce fluent language, it may fail to capture pragmatic, cultural, and situational nuances (Godwin-Jones, 2024). Such limitations are particularly relevant to English-mediated intercultural business communication, where politeness, persuasion, emotion, credibility, humor, complaint handling, and relationship maintenance often depend on subtle linguistic and cultural cues. Accordingly, the educational value of AI-enhanced social media depends not merely on access to AI functions, but also on how students use these functions for meaningful intercultural communication and whether their English proficiency enables deeper engagement.

AI-enhanced social media refers to general-purpose social media environments in which embedded or connected AI functions support intercultural exposure, comprehension, expression, feedback, and interpretation. These functions are algorithmic recommendation, machine translation, automatic subtitles, AI-assisted writing, AI-based explanation, content filtering, sentiment cues and generative language support (Huang et al., 2023). AISM refers to general-purpose social media environments that offer access to user-generated content and online communities and influence the intercultural content to which students are exposed, how they access multilingual information, how they formulate or modify responses, and how they interpret unfamiliar cultural meanings. According to the concept of communication affordances, the functions of these platforms should be regarded as relational possibilities for action rather than as fixed technological attributes (Evans et al., 2017). In light of the above, AISM can be viewed as a digitally mediated communication space that combines the functions of social media with AI-powered language and culture assistance.

Social media provides students with opportunities for digitally mediated intercultural communication by allowing them to observe, interpret, react to and reflect on culturally diverse communication in daily life on these platforms. Research on the affordances of social media has found that visibility, persistence, editability and association all affect how users obtain information, form relationships and join communication networks (Treem and Leonardi, 2013). The above affordances provide opportunities for Chinese business students to participate in overseas consumer discussions, learn from different cultures, ask and answer questions in the comment area, and explore how meaning is generated in platform-based business communication. At the same time, there are limitations to the affordances of social media: algorithmic filtering may limit exposure, and online interactions may remain relatively superficial, and translation support can facilitate comprehension but may obscure pragmatic nuance. Reviews of technology-supported cross-cultural learning thus indicate that digital tools can promote intercultural learning only when they are incorporated into meaningful interaction, task design and reflection (Shadiev et al., 2021; Shadiev et al., 2024). Recently, research on AI language learning has also pointed out the advantages of adaptive feedback and learner engagement, but it has not fully explained how AI embedded in social media platforms is linked to ICC through engagement, identity integration and English proficiency (Huang et al., 2023). Therefore, although engagement, negotiation of cultural identity and language proficiency have been studied in various intercultural and language-learning situations, their joint roles among Chinese business students using AI-enhanced social media remain unexplored.

Therefore, this paper has three aims. First, it investigates the relationship between AI-enhanced social media affordances and intercultural communication competence for Chinese business students. Second, it examines whether intercultural interaction engagement and cultural identity integration mediate this association separately and sequentially. Third, it examines whether English proficiency functions as a linguistic enabling condition that moderates the relationship between AI-enhanced social media affordances and intercultural interaction engagement, and subsequently the indirect associations with ICC. Integrating social media affordance theory, technology-mediated intercultural learning and ICC research, this paper seeks to explore whether AI-enhanced social media affordances are positively associated with intercultural communication competence (ICC) and, if so, how and under what linguistic circumstances such a correlation occurs among Chinese business students.

## 2 Literature review

### 2.1 Theoretical background

The resource-based view and the theory of dynamic capabilities help explain how external resources are converted into actual capabilities through situated deployment and internal capability development (Barney, 1991; Grant, 1991; Teece et al., 1997) as resource-conversion lenses. Although these perspectives originated in strategic management, they are only applied here as supporting lenses to understand how Chinese business students can transform external digital resources and internal learner resources into professional communicative ability. The main theoretical foundations of the study are affordance theory, ICC theory, online intercultural learning, identity negotiation, and communicative language ability. AI-enhanced social media affordances refer to external digital resources in this framework; intercultural interaction engagement is the process by which these resources are realized; cultural identity integration is an internal identity resource; and English proficiency is an internal linguistic resource that influences how students use AI-supported platform functions.

Communicative competence includes grammatical, sociolinguistic, discourse, and strategic dimensions and therefore extends beyond linguistic form alone (Canale and Swain, 1980). Byram’s (1997) framework further emphasizes interpreting, relating, discovery, interaction, and critical cultural awareness, while Deardorff (2006) conceptualizes intercultural competence through attitudes, knowledge, skills, and effective and appropriate communication. In the present context, ICC therefore refers to students’ perceived capacity to understand, interpret, and respond appropriately to culturally diverse business communication, including consumer expectations, brand narratives, service evaluations, workplace discussions, platform comments, and professional online interactions. This context also requires pragmatic and interactional competence, including turn-taking, repair, contextual adaptation, politeness, persuasion, and situated meaning-making (Galaczi and Taylor, 2018; Roever and Kasper, 2018).

Affordance theory adds a new component to the resource-capability theory and considers external digital resources to be opportunities for action. In the study of communication affordances, they are not considered to be fixed technological attributes but rather as relational possibilities that arise from the interaction of users, technology and the social environment (Evans et al., 2017). This is necessary because the current study does not consider AI as an independent learning system; rather, it explores AI-enhanced social media as an ecosystem of platforms that incorporate algorithmic recommendations, machine translation, automatic subtitles, AI-assisted writing, AI-based explanations, content filtering and sentiment cues in daily intercultural and business communication. Research on the affordances of social media shows that visibility, persistence, editability and association influence how people are informed about problems, build networks, and conduct professional and organizational work (Treem and Leonardi, 2013). Social media is widely used for promotion and business communication to attract consumers and enhance brand recognition among the public (Appel et al., 2020). Therefore, AI-enhanced social media affordances are conceived of as external digital resources that can support intercultural exposure, understanding, expression, feedback and cultural interpretation. However, these affordances are not inherently good: algorithmic visibility may increase exposure but decrease attention; translation can help people understand but may lose pragmatic connotations; and AI-assisted expression can enhance self-confidence without ensuring suitability.

The proposed framework assumes that the association between AI-enhanced social media affordances and ICC is conditioned by students’ engagement, identity integration, and linguistic resources. Research on online intercultural learning shows that digital interaction can support ICC through interpretation, comparison, dialogue and reflection, rather than passive observation (Chun, 2011). Research on virtual exchange also indicates organized cross-cultural interaction in digital spaces (O’Dowd, 2018), and at the same time, reviews of technology-supported cross-cultural learning show that technology can help achieve this goal when combined with meaningful communication and reflective task design (Shadiev et al., 2021; Shadiev et al., 2024). Therefore, intercultural interaction engagement in this study is understood as the way students actualize the AI-enhanced social media affordances in content-based, interactive, reflective, and relational forms. Cultural identity integration also helps explain how intercultural interaction relates to students’ sense of self and communicative position. In accordance with the theory of identity negotiation, cross-cultural communication refers to the process of negotiating one’s own identity and sense of cultural belonging in different cultures (Ting-Toomey, 2005), and research on bicultural identity integration suggests that individuals vary in whether they perceive different cultural frameworks as compatible or conflicting (Benet-Martínez and Haritatos, 2005). English proficiency also affects whether students will use AI translation, subtitles, writing support and feedback mainly for surface-level comprehension or for deeper evaluation, pragmatic refinement and active participation. Research on AI language education also shows that intelligent tools are most effective when learners actively evaluate and apply them in meaningful communicative activities (Huang et al., 2023). Engagement, identity integration, and English proficiency are therefore theorized to shape how students actualize these digital resources.

### 2.2 Hypothesis development

#### 2.2.1 AI-enhanced social media affordances, intercultural interaction engagement, and cultural identity integration

AI-enhanced social media affordances (AIESMA) refer to students’ perceived opportunities to use AI-assisted social media functions for intercultural exposure, multilingual comprehension, English-mediated expression, pragmatic feedback and cultural interpretation in business-related platform communication (Evans et al., 2017). AIESMA are embedded in concrete platform experiences where algorithmic recommendation expands access to international consumer reviews, brand narratives, workplace discussions, livestreams, service complaints, and culturally diverse comments (Bartoloni et al., 2024). Machine translation and automatic subtitles support students’ comprehension of multilingual posts, videos, and replies, while AI-assisted writing and AI-based explanation facilitate English response drafting, pragmatic interpretation, and cultural meaning-making (Huang et al., 2023). Generative AI research further indicates that AI-supported language tools can create opportunities for feedback, revision, interaction, and learner agency when students use them critically rather than passively (Kohnke et al., 2023). These AI-supported affordances are therefore expected to strengthen intercultural interaction engagement (IIE), because technology-supported cross-cultural learning is most effective when learners participate in communication, comparison, and reflection rather than merely access information (Shadiev et al., 2024). AIESMA are also expected to support cultural identity integration (CII), because repeated exposure to international evaluations of Chinese products, brands, service practices, workplace norms, and cultural values can stimulate self–other comparison and cultural self-positioning (Zhou et al., 2024). Identity negotiation theory explains that intercultural communication involves the negotiation of cultural memberships, self-images, and relational meanings across cultural boundaries (Ting-Toomey, 2005). Bicultural identity integration research further suggests that individuals differ in whether they perceive different cultural frames as compatible or oppositional (Benet-Martínez and Haritatos, 2005). Online intercultural learning research also shows that digital interaction can support intercultural competence when learners connect cultural comparison with reflective participation (Zuo, 2025). Accordingly, the following hypotheses are proposed:

*H1:* AI-enhanced social media affordances are positively associated with intercultural interaction engagement among Chinese business students.

*H2:* AI-enhanced social media affordances are positively associated with cultural identity integration among Chinese business students.

#### 2.2.2 Intercultural interaction engagement, cultural identity integration, and intercultural communication competence

Intercultural communication competence (ICC) refers to students’ ability to understand, interpret, and respond appropriately and effectively to culturally diverse everyday and business-related communication in English-mediated social media environments (Deardorff, 2006). Communicative competence theory emphasizes that effective language use involves grammatical, sociolinguistic, discourse, and strategic competence rather than linguistic accuracy alone (Canale and Swain, 1980). Recent China-related ICC research further shows that cross-cultural communicative competence is increasingly important for undergraduate English education, global communication, and professional participation (Zhou et al., 2024). IIE can provide opportunities for students to observe authentic interactions, compare how similar meanings are expressed across cultures, identify underlying cultural values, and adapt their communication to different online contexts (Zuo, 2025). Technology-supported cross-cultural learning research also indicates that communication-rich and reflection-oriented digital activities are more closely related to intercultural learning than passive exposure to foreign-language or foreign-culture materials (Shadiev et al., 2024). CII is expected to support ICC because students who integrate Chinese cultural grounding with global business participation are more likely to communicate with openness, flexibility, pragmatic appropriateness, and intercultural sensitivity (Zhou et al., 2024). Accordingly, the following hypotheses are proposed:

*H3:* Intercultural interaction engagement is positively associated with cultural identity integration among Chinese business students.

*H4:* Intercultural interaction engagement is positively associated with intercultural communication competence among Chinese business students.

*H5:* Cultural identity integration is positively associated with intercultural communication competence among Chinese business students.

#### 2.2.3 Associations between AI-enhanced social media affordances and intercultural communication competence

AI environments support multiple languages for translation, recommendations expand exposure to foreign materials, and AI-assisted writing can support language production, but none of these functions automatically guarantee pragmatic appropriateness or cultural sensitivity (Godwin-Jones, 2024). Recently, AI language education research has found that intelligent tools are most effective when students use them actively for participation, feedback-oriented learning and communication practice (Huang et al., 2023). IIE therefore represents a practice-based explanatory link between AIESMA and ICC, because AI-supported access, comprehension, expression, feedback, and interpretation can facilitate intercultural content engagement, dialogue, perspective comparison, and communication strategy practice (Kohnke et al., 2023). CII represents an identity-based explanatory link between AIESMA and ICC, because AI-supported exposure to diverse cultural frames can encourage students to reflect on how Chinese identity can be articulated within global business communication (Zhou et al., 2024). Online intercultural learning research further suggests that digital intercultural competence develops more effectively when learners engage in interaction, reflection, and comparison across cultural boundaries (Zuo, 2025).

*H6:* AI-enhanced social media affordances are positively associated with intercultural communication competence among Chinese business students.

*H7:* Intercultural interaction engagement mediates the relationship between AI-enhanced social media affordances and intercultural communication competence.

*H8:* Cultural identity integration mediates the relationship between AI-enhanced social media affordances and intercultural communication competence.

*H9:* AI-enhanced social media affordances are indirectly associated with intercultural communication competence through the sequential mediation of intercultural interaction engagement and cultural identity integration.

#### 2.2.4 English proficiency as a moderator

English proficiency (EP) refers to students’ ability to understand, evaluate, and produce English-mediated communication in AI-enhanced social media environments, which is consistent with the communicative language ability perspective (Bachman and Palmer, 1996). EP is conceptually different from ICC because ICC also includes cultural interpretation, pragmatic appropriateness, intercultural sensitivity, multimodal cue interpretation, and interactional effectiveness (Zhou et al., 2024). EP can condition how students transform AIESMA into IIE because international consumer communication, professional networking, and global business discourse are frequently mediated through English (Bartoloni et al., 2024). AI translation, automatic subtitles and AI-assisted writing can serve as compensatory scaffolds for lower-proficiency students to reduce the basic comprehension and expression gap (Huang et al., 2023). For higher-proficiency students, the same AI-supported affordances are expected to have an amplifying function because these students are more capable of checking AI output, identifying cultural or pragmatic mismatch, adapting English expressions, and participating in deeper intercultural interaction (Godwin-Jones, 2024). This moderating effect is also expected to extend to the sequential indirect association from AIESMA to ICC through IIE and CII, because stronger English proficiency can make AI-supported intercultural engagement more reflective, interactive, and identity-relevant (Zuo, 2025). In contrast, students with weaker EP can gain access to intercultural content through AI support, but the transformation from access to engagement, identity integration, and competence is expected to be weaker (Shadiev et al., 2024). Accordingly, the following hypotheses are proposed:

*H10:* English proficiency positively moderates the relationship between AI-enhanced social media affordances and intercultural interaction engagement, such that the association is stronger among students with higher English proficiency.

*H11:* English proficiency positively moderates the sequential indirect association between AI-enhanced social media affordances and intercultural communication competence through intercultural interaction engagement and cultural identity integration, such that the indirect association is stronger among students with higher English proficiency.

The theoretical framework diagram is shown in Figure 1.

**FIGURE 1:**
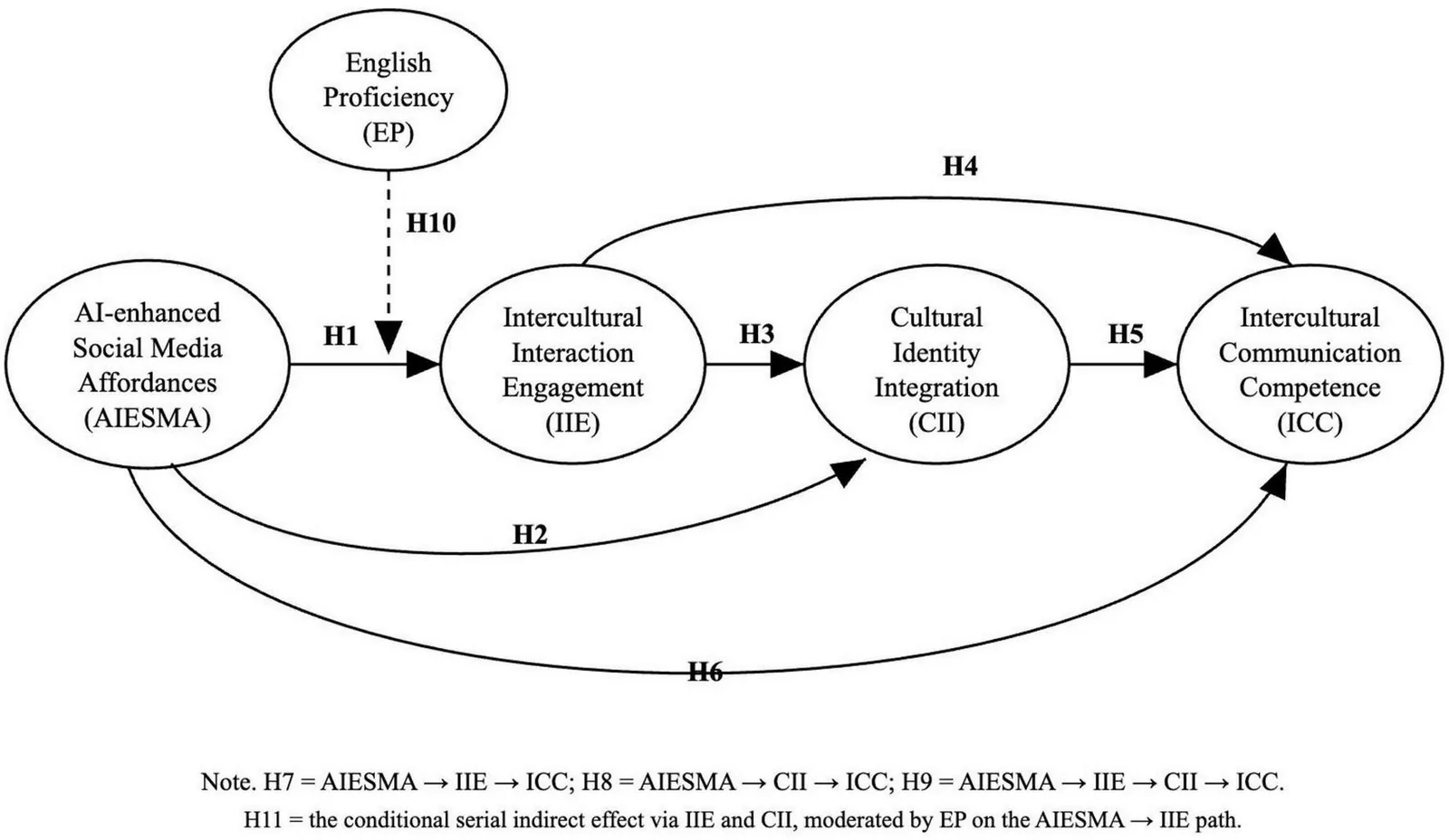
Theoretical framework.

## 3 Research method

### 3.1 Research design

This study used a two-study design to develop and test a model linking AI-enhanced social media affordances to intercultural communication competence among Chinese business students. The two studies served different but connected purposes. Study 1 focused on instrument development and preliminary validation. It refined the measurement instrument through item generation, expert review, cognitive interviews, exploratory factor analysis, reliability assessment, and preliminary validity testing. Study 2 used an independent sample to test the full theoretical model through partial least squares structural equation modeling. Although some recruitment sites overlapped, Study 1 and Study 2 drew independent participant pools through separate survey links, recruitment waves, and screening procedures. Duplicate responses were screened using response timestamps, IP/device information where available, and demographic consistency checks, and no respondent from Study 1 was retained in the Study 2 dataset.

The study protocol received minimal-risk expedited review and approval from the Ethics Committee of the Sustainable Development Research Centre (IRB No. SDRC-EC-HSS-2026-02-005) on 5 February 2026. The committee reviewed the research protocol, questionnaire, participant information sheet, and consent materials. Before entering the questionnaire, all adult participants viewed an electronic informed-consent page describing the study, confirmed that they had read the information, and voluntarily agreed to participate. Participation was voluntary and anonymous, and participants could discontinue before submitting the questionnaire.

### 3.2 Study 1: instrument development and preliminary validation

Study 1 was designed as the instrument development and preliminary validation stage of the two-study design. Its purpose was not to test the full hypothesized structural model, but to refine and preliminarily validate the measurement instrument before the main model test in Study 2. This stage was necessary because AI-enhanced social media affordances had to be measured as a perceived set of action possibilities embedded in specific AI-supported social media experiences, rather than as a general perception of technological usefulness. It was also necessary because intercultural communication competence was contextualized in business-related, English-mediated, and AI-supported social media communication.

#### 3.2.1 Data collection and descriptive statistics

Study 1 collected online survey data from Chinese undergraduate and postgraduate students enrolled in business-related programs from 16 February to 20 March 2026. Participants were recruited from six universities in Beijing, Shanghai, Guangzhou, Wuhan, Chengdu, and Chongqing. Purposive and convenience sampling were used to recruit students whose academic backgrounds and platform-use experiences met the study conditions. Recruitment notices were distributed through business-school course groups, business English and international business course communities, e-commerce and digital trade classes, student associations, university mailing lists, and WeChat and QQ groups. Recruitment covered undergraduate and postgraduate students in international business, e-commerce, digital trade, marketing, finance, accounting, tourism management, hospitality management, logistics, entrepreneurship, and business English. Recruitment notices directed interested students to an electronic participant-information and consent page before they entered the questionnaire.

Screening questions were included at the beginning of the questionnaire to verify that participants met the inclusion conditions. Eligible respondents were 18 years old or older, were Chinese university students majoring in business-related fields, and had experience using AI-enhanced social media functions in English-mediated, multilingual, or intercultural platform environments. Examples of such functions included algorithm-based recommendations, machine translation, automatic subtitles, AI-assisted writing, and AI-based explanations.

A total of 436 responses were collected in Study 1. After data screening, 36 responses were excluded because they did not meet the screening questions, contained too many missing values, showed a straight-lining response pattern, or had unrealistically short completion times. Finally, 400 valid responses were retained, with a valid response rate of 91.7%. The planned valid sample was approximately 400, primarily to support multidimensional exploratory factor analysis of the 57-item pool and interpretable factor recovery; the resulting ratio of approximately seven respondents per initial item was treated as a secondary adequacy consideration. KMO statistics, strong factor loadings, and theoretically interpretable factor solutions provided empirical support for sample adequacy.

Table 1 shows the demographic and background information of the respondents in Study 1. Among the 400 valid respondents, 171 were male (42.8%) and 229 were female (57.3%). Most respondents were aged 21–23 (59.0%). Undergraduate students accounted for 87.0% of the sample (*n* = 348), while postgraduate students accounted for 13.0% (*n* = 52). Respondents represented a range of business-related majors, including international business, e-commerce and digital trade, marketing, finance and accounting, and tourism and hospitality.

**TABLE 1:** Demographic and background profile of Study 1 respondents.

Characteristic	Category	Frequency	Percentage
Gender	Male	171	42.80%
	Female	229	57.30%
Age	18–20	112	28.00%
	21–23	236	59.00%
	24 or above	52	13.00%
Education level	Undergraduate	348	87.00%
	Postgraduate	52	13.00%
Year of study	Year 1	74	18.50%
	Year 2	102	25.50%
	Year 3	111	27.80%
	Year 4 or above	61	15.30%
	Postgraduate	52	13.00%
Major	International business	88	22.00%
	E-commerce or digital trade	79	19.80%
	Marketing	71	17.80%
	Finance or accounting	67	16.80%
	Tourism or hospitality	53	13.30%
	Other business-related majors	42	10.50%
Self-reported English proficiency	Low	69	17.30%
	Moderate	205	51.30%
	High	126	31.50%
Social media use frequency	Several times a week	45	11.30%
	Once a day	91	22.80%
	Several times a day	264	66.00%
Experience with AI-supported social media functions	Less than 6 months	77	19.30%
	6 months to 1 year	123	30.80%
	1–2 years	134	33.50%
	More than 2 years	66	16.50%
Most frequently used AI-supported function	Algorithmic recommendation	89	22.30%
	Machine translation	104	26.00%
	Automatic subtitles	70	17.50%
	AI-assisted writing	76	19.00%
	AI-based explanation	61	15.30%

*N* = 400. Percentages may not sum to exactly 100 because of rounding.

#### 3.2.2 Measurement development

Study 1 followed a staged scale-development procedure because the construct of AI-enhanced social media affordances is new and context-specific, and because intercultural communication competence was contextualized for business-relevant, English-mediated, and AI-supported social media communication. The item-generation process followed established recommendations for survey measure development, including construct definition, item generation, expert review, cognitive interviews, and preliminary psychometric testing (Hinkin, 1998). The initial item pool included 18 AIESMA items, 10 IIE items, 7 CII items, 5 EP items, and 17 ICC items. All initial self-report items used a seven-point Likert scale ranging from 1 = strongly disagree to 7 = strongly agree. The initial items were then reviewed and refined through expert review, cognitive interviews, and preliminary validation procedures. The full item pool, retained items, deleted items, and item-retention rationales are reported in Supplementary File 1, while expert-review and cognitive-interview refinement details are reported in Supplementary File 2.

The starting pool of items was 57. The AIESMA items were newly developed from affordance theory and communication-affordance research, in which affordances refer to relational action possibilities arising from the interaction among users, technologies, and social contexts (Evans et al., 2017). Items for intercultural interaction engagement were contextually developed with reference to online intercultural learning and the view that digital intercultural learning requires interpretation, comparison, dialogue, and reflection rather than passive exposure alone (Chun, 2011). These items focused on students’ content-based, interactive, reflective, and relational participation in online intercultural communication. Cultural identity integration items were adapted and contextualized with reference to research on bicultural identity integration and intercultural identity (Benet-Martínez and Haritatos, 2005). English proficiency items were contextually developed with reference to the communicative language ability perspective (Bachman and Palmer, 1996) and measured students’ perceived ability to understand, evaluate, and produce English in AI-enhanced social media environments. ICC items were contextually developed with reference to intercultural competence frameworks, especially the view that intercultural competence includes attitudes, knowledge, skills, and effective and appropriate communication (Deardorff, 2006).

#### 3.2.3 Item refinement and preliminary validation analysis

A reduced set of initial items was selected based on expert judgment and cognitive interviews prior to exploratory factor analysis. Eight experts reviewed the initial items. The panel included two applied linguistics scholars, two intercultural communication scholars, two business English or international business communication instructors, and two educational technology researchers. Using a four-point content-validity scale, the experts rated item relevance and also evaluated conceptual fit, clarity, contextual suitability, redundancy, and construct representation. Items with an item-level content validity index (I-CVI) of at least .78 were retained unless qualitative comments identified a substantive conceptual or wording problem (Polit et al., 2007). Items below this threshold or lacking conceptual fit, clarity, or distinctiveness were revised or excluded. Detailed decisions are reported in Supplementary File 2.

Cognitive interviews were then conducted with 24 Chinese business students from different majors and years of study. During the interviews, participants read items, explained what they understood each item to mean, identified ambiguous expressions, and judged whether the wording matched their actual contexts of AI-enhanced social media use. This step clarified the terms pragmatic feedback, multimodal cue interpretation, AI-supported cultural interpretation, and business-related intercultural communication. Based on the expert review and cognitive interview feedback, six ambiguous or overlapping items were excluded before exploratory factor analysis, and 51 retained items were used for exploratory factor analysis, reliability testing, and preliminary validity assessment.

Exploratory factor analyses were performed separately for AIESMA, IIE, CII, EP, and ICC on the 51 retained items. Principal axis factoring with Promax oblique rotation was selected because the constructs and their dimensions were expected to be correlated. Factor retention was determined using parallel analysis, scree-plot inspection, and theoretical interpretability (Watkins, 2018). Item retention required a primary loading of at least 0.50, a communality of at least 0.40, and theoretical interpretability. For multidimensional constructs, cross-loadings also had to remain below 0.30, with a difference of at least 0.20 between the primary and next-highest loading. For unidimensional constructs, the cross-loading rule was considered only when an interpretable alternative factor was present and was not applied mechanically. All 51 items met the applicable criteria, and no item was deleted after exploratory factor analysis. Primary EFA pattern-matrix loadings for all 51 retained items are reported in Supplementary File 3.

### 3.3 Study 2: main PLS-SEM model test

Study 2 used an independent sample of Chinese business students to test the hypotheses. Study 1 focused on indicator development and initial validation; Study 2 employed the validated indicators from Study 1 to test the measurement and structural models. The goal of Study 2 was to evaluate the direct associations, indirect associations, sequential indirect associations, moderation, and moderated mediation proposed in H1–H11.

#### 3.3.1 Data collection and descriptive statistics

Study 2 collected online data from an independent participant pool from 15 April to 31 May 2026. A separate recruitment wave covered eight universities in Beijing, Shanghai, Guangzhou, Shenzhen, Wuhan, Chengdu, Chongqing, and Xi’an. Purposive and convenience sampling were used to recruit students whose academic programs and platform use matched the research context. Recruitment notices were distributed through business schools, business English programs, e-commerce and digital trade courses, course-related online communities, student associations, university mailing lists, and WeChat and QQ groups. The inclusion criteria were the same as in Study 1. Respondents were over 18 years old, were Chinese university students in business-related undergraduate or postgraduate programs, and had used AI-enhanced social media functions in English-mediated, multilingual, or intercultural platform environments.

A total of 882 responses were collected in Study 2. After data screening, 82 responses were excluded because they did not meet the screening criteria, contained too many missing values, showed straight-lining patterns, failed attention checks, or had unrealistically short completion times. Finally, 800 valid responses were retained for the main model test, and the valid response rate was 90.7%. As a prospective planning rationale, the inverse square root approach with α = 0.05, power = 0.80, and a minimum substantively meaningful standardized path coefficient of .10 indicated a minimum sample of approximately 619 (Kock and Hadaya, 2018). The planned target of 800 valid responses also supported the mediation, moderation, and robustness analyses. Table 2 shows the demographics and AI-enhanced platform-use patterns of the respondents in Study 2.

**TABLE 2:** Study 2 sample and AI-enhanced platform-use profile.

Characteristic	Category	Frequency	Percentage
Valid responses	Total	800	100.00%
Gender	Male	346	43.30%
	Female	454	56.80%
Education level	Undergraduate	694	86.80%
	Postgraduate	106	13.30%
Year of study	Year 1	136	17.00%
	Year 2	228	28.50%
	Year 3	268	33.50%
	Year 4 or above	62	7.80%
	Postgraduate	106	13.30%
Major	International business	176	22.00%
	E-commerce or digital trade	158	19.80%
	Marketing	137	17.10%
	Finance or accounting	130	16.30%
	Tourism or hospitality	108	13.50%
	Logistics, entrepreneurship, or other business majors	91	11.40%
AI-supported function use	Algorithmic recommendation	754	94.30%
	Automatic subtitles	612	76.50%
	Machine translation	553	69.10%
	AI-assisted writing	371	46.40%
	AI-based explanation	349	43.60%
Platform exposure	Domestic-platform dominant	526	65.80%
	Foreign-platform exposed	274	34.30%
English background	Reported English test indicators available	476	59.50%
International background	Overseas or international experience	126	15.80%

*N* = 800. Platform functions were not mutually exclusive. Percentages may not sum to exactly 100 because of rounding.

As shown in Table 2, the Study 2 sample was appropriate for testing the hypothesized model. Respondents represented a broad range of business-related majors and showed substantial exposure to AI-supported social media functions. The inclusion of both domestic-platform dominant users and foreign-platform exposed users supported the relevance of the sample for examining English-mediated and intercultural communication in platform environments. Reported English test scores were available for a subset of respondents and were used for a supplementary test-based relative proficiency robustness check.

#### 3.3.2 Measures used in Study 2

The final Study 2 questionnaire was based on the measurement instrument developed and preliminarily validated in Study 1. Only the items retained after expert review, cognitive interviews, exploratory factor analysis, reliability assessment, and preliminary validity testing were used in the main model test. All self-report items were measured using a seven-point Likert scale ranging from 1 = strongly disagree to 7 = strongly agree.

AIESMA was measured as a multidimensional construct with five lower-order dimensions: algorithmic cultural exposure, AI-mediated comprehension support, AI-assisted expression support, AI-enabled pragmatic feedback, and AI-supported cultural interpretation. Each dimension was measured with three items. These dimensions captured students’ perceived opportunities to use AI-enhanced social media functions for intercultural exposure, multilingual understanding, English-mediated expression, pragmatic feedback, and interpretation of culturally embedded meanings in business-related platform communication.

IIE consisted of four dimensions, with two items measuring each dimension and eight items in total. The four dimensions were content engagement, interactive engagement, reflective engagement, and relational engagement. The items examined the degree to which students engaged with culturally diverse social media content, participated in intercultural platform interaction, reflected on cultural meanings, and attended to intercultural relationships and communities.

CII was measured using six items adapted and contextualized from identity integration and intercultural identity research. The items assessed students’ sense of Chinese cultural identity in global business contexts, their perceived compatibility between Chinese and foreign cultural frameworks, and their perceived ability to express Chinese perspectives in culturally appropriate and internationally understandable ways.

Self-reported EP was operationalized as a five-item latent construct measuring students’ perceived ability to comprehend, evaluate, and produce English in AI-enhanced social media communication. Reported scores from CET-4, CET-6, IELTS, TOEFL, and BEC, where available, were collected as background information and used for a supplementary test-based relative proficiency robustness check rather than being included in the main latent construct.

ICC was operationalized as students’ self-reported perceptions of their intercultural communication competence. It was modeled as a multidimensional construct of business-relevant ICC. The six dimensions were cultural understanding, intercultural sensitivity, communication strategies, pragmatic appropriateness, multimodal cue interpretation, and interactional effectiveness. The contexts included consumer comments, brand stories, professional talks, cross-border e-commerce interactions, platform comment sections, live-streaming sessions, and everyday intercultural scenarios. Contextualization ensured that the measure represented perceived business-relevant, platform-based communication competence rather than general cultural awareness or general English proficiency.

The final item wording used in Study 2 is reported in Supplementary File 1, and item-level standardized loadings are reported in Supplementary File 4.

#### 3.3.3 Data analysis procedure

Study 2 tested the hypothesized model using partial least squares structural equation modeling. PLS-SEM was appropriate because the model included a multidimensional higher-order construct, sequential mediation, moderation, moderated mediation, and a prediction-oriented theoretical framework (Hair et al., 2022; Sarstedt et al., 2016). The analysis followed a two-step procedure. First, the measurement model was assessed. Second, the structural model and hypothesis tests were evaluated. For the moderation analysis, a two-stage PLS procedure was used (Becker et al., 2012; Sarstedt et al., 2019). In the first stage, AIESMA higher-order construct scores and EP scores were estimated. In the second stage, their interaction term was used to predict IIE. Statistical inference used 5,000 bootstrap resamples, and simple slopes were evaluated at one standard deviation below the mean, at the mean, and at one standard deviation above the mean of EP.

## 4 Results

### 4.1 Study 1: scale development and preliminary validation

Study 1 refined and preliminarily validated the measurement instrument before the main model test in Study 2. The initial item pool contained 57 items, including 18 items for AI-enhanced social media affordances, 10 items for intercultural interaction engagement, 7 items for cultural identity integration, 5 items for English proficiency, and 17 items for intercultural communication competence. After expert review and cognitive interviews, six items were removed because of conceptual overlap, ambiguous wording, weak business-context fit, or difficulty in student interpretation. The deleted items included three AIESMA items, two IIE items, and one CII item. No EP or ICC items were removed at this stage. As shown in Table 3, the final Study 1 instrument retained 51 items, including 15 AIESMA items, 8 IIE items, 6 CII items, 5 EP items, and 17 ICC items. The full item-level wording and deletion decisions are reported in Supplementary File 1, expert-review and refinement details in Supplementary File 2, Study 1 EFA loadings in Supplementary File 3, and Study 2 standardized loadings in Supplementary File 4.

**TABLE 3:** Study 1 item-retention summary.

Construct	Dimensions retained	Initial items	Deleted items	Retained items	Main reason for deletion
AIESMA	5	18	3	15	Conceptual overlap, ambiguous wording, weak contextual fit
IIE	4	10	2	8	Conceptual overlap and unclear wording
CII	1	7	1	6	Overlap with general cultural pride
EP	1	5	0	5	No item deleted
ICC	6	17	0	17	No item deleted
Total	17	57	6	51	Not applicable

AIESMA, AI-enhanced social media affordances; IIE, intercultural interaction engagement; CII, cultural identity integration; EP, English proficiency; ICC, intercultural communication competence. Full item wording is reported in Supplementary File 1, and item-level diagnostics are reported in Supplementary Files 3 and 4.

Exploratory factor analysis was then conducted on the 51 retained items. Principal axis factoring with Promax oblique rotation was used because the dimensions were theoretically expected to be correlated. The retained structure was consistent with the proposed measurement framework. Parallel analysis and scree plot inspection supported five factors for AIESMA, four factors for IIE, six factors for ICC, and one factor each for CII and EP. AIESMA retained five dimensions: algorithmic cultural exposure, AI-mediated comprehension support, AI-assisted expression support, AI-enabled pragmatic feedback, and AI-supported cultural interpretation. IIE retained four dimensions: content engagement, interactive engagement, reflective engagement, and relational engagement. ICC retained six dimensions: cultural understanding, intercultural sensitivity, communication strategies, pragmatic appropriateness, multimodal cue interpretation, and interactional effectiveness. CII and EP were retained as single-dimensional constructs.

As shown in Table 4, factorability was acceptable for all retained constructs. KMO values ranged from 0.879 to 0.952, and Bartlett’s tests of sphericity were significant for all constructs. The retained factors explained between 65.21 and 74.92% of the variance across constructs. EFA loading ranges were above commonly accepted thresholds, and Cronbach’s alpha values ranged from 0.887 to 0.948, indicating acceptable preliminary reliability. Alpha values in Table 4 refer to construct-level preliminary internal consistency for the retained item sets; dimension-level reliability was further assessed in the Study 2 measurement model. Composite reliability and AVE were assessed in the Study 2 measurement model rather than in Study 1, because Study 1 served as the exploratory and preliminary validation stage.

**TABLE 4:** Study 1 EFA and preliminary reliability summary.

Construct	Dimensions retained	Items retained	KMO	Bartlett p	Variance explained	Loading range	Alpha
AIESMA	5	15	0.948	< 0.001	73.84%	0.742–0.914	0.934
IIE	4	8	0.902	< 0.001	70.46%	0.758–0.921	0.91
CII	1	6	0.895	< 0.001	65.21%	0.765–0.861	0.901
EP	1	5	0.879	< 0.001	68.77%	0.781–0.852	0.887
ICC	6	17	0.952	< 0.001	74.92%	0.751–0.932	0.948

EFA used principal axis factoring with Promax oblique rotation. Alpha = Cronbach’s alpha. Alpha values refer to construct-level preliminary internal consistency for the retained item sets. Primary EFA pattern-matrix loadings for all 51 retained items are reported in Supplementary File 3.

The Study 1 results supported the transition to Study 2. The retained 51-item instrument was theoretically interpretable and showed acceptable preliminary psychometric quality. KMO statistics, strong factor loadings, and theoretically interpretable factor solutions provided empirical support for sample adequacy. Therefore, the retained items were used as the basis for the final Study 2 questionnaire.

### 4.2 Study 2: descriptive statistics and correlations

Study 2 used an independent sample of 800 Chinese business students to test the measurement and structural models. As shown in Table 5, the descriptive statistics, normality indicators, and correlations among the main constructs were within acceptable ranges, and all reported correlations were statistically significant.

**TABLE 5:** Descriptive statistics, normality indicators, and correlations.

Construct	M	SD	Skewness	Kurtosis	AIESMA	IIE	CII	EP	ICC
AIESMA	5.31	0.84	−0.396	0.274	–	–	–	–	–
IIE	5.08	0.9	−0.331	0.218	0.602				
CII	5.2	0.8	−0.284	0.162	0.322	0.437			
EP	4.96	0.88	−0.211	0.097	0.342	0.413	0.374		
ICC	5.24	0.77	−0.352	0.246	0.51	0.6	0.615	0.421	

Correlations are reported using construct scores. AIESMA correlations are based on higher-order construct scores. All reported correlations were significant at p < 0.001. Fornell-Larcker discriminant-validity evidence is reported in Supplementary File 6.

### 4.3 Measurement model assessment

The Study 2 measurement model was assessed using the final measurement structure developed and preliminarily validated in Study 1. Table 6 reports reliability and convergent validity at the dimension level, while full item-level standardized loadings are reported in Supplementary File 4.

**TABLE 6:** Study 2 measurement model reliability and convergent validity.

Construct	Dimension	Items	Loading range	Alpha	rho_A	CR	AVE
AIESMA	Algorithmic cultural exposure	3	0.828–0.899	0.884	0.886	0.9	0.751
	AI-mediated comprehension support	3	0.821–0.892	0.872	0.875	0.895	0.74
	AI-assisted expression support	3	0.840–0.911	0.895	0.897	0.91	0.772
	AI-enabled pragmatic feedback	3	0.836–0.906	0.887	0.889	0.907	0.766
	AI-supported cultural interpretation	3	0.853–0.918	0.904	0.906	0.921	0.795
IIE	Content engagement	2	0.914–0.936	0.846	0.848	0.922	0.856
	Interactive engagement	2	0.921–0.946	0.876	0.878	0.931	0.872
	Reflective engagement	2	0.918–0.941	0.861	0.863	0.927	0.864
	Relational engagement	2	0.916–0.938	0.852	0.854	0.924	0.86
CII	Cultural identity integration	6	0.792–0.866	0.914	0.916	0.931	0.691
EP	English proficiency	5	0.814–0.861	0.898	0.9	0.923	0.705
ICC	Cultural understanding	3	0.864–0.923	0.861	0.863	0.923	0.803
	Intercultural sensitivity	3	0.872–0.931	0.879	0.881	0.931	0.815
	Communication strategies	3	0.881–0.936	0.888	0.89	0.936	0.829
	Pragmatic appropriateness	3	0.886–0.943	0.903	0.905	0.939	0.839
	Multimodal cue interpretation	2	0.889–0.941	0.874	0.876	0.913	0.839
	Interactional effectiveness	3	0.875–0.934	0.886	0.888	0.934	0.821

Alpha, Cronbach’s alpha; CR, composite reliability; AVE, average variance extracted. AIESMA dimensions were assessed as reflective lower-order dimensions and were then used as indicators of the higher-order formative AIESMA construct. Full item-level standardized loadings are reported in Supplementary File 4.

Discriminant validity among the reflective constructs and higher-order construct scores was assessed using HTMT and the Fornell-Larcker criterion. Because AIESMA was modeled as a reflective-formative higher-order construct, HTMT was used as an additional diagnostic rather than as the sole basis for evaluating discriminant validity. Table 7 shows that all HTMT values were below the conservative threshold of 0.85. The highest HTMT value appeared between IIE and ICC, which was conceptually reasonable because intercultural engagement and intercultural communication competence are closely related. However, the value remained below the level that would indicate construct redundancy.

**TABLE 7:** HTMT discriminant validity matrix.

Construct pair	HTMT
AIESMA and IIE	0.694
AIESMA and CII	0.446
AIESMA and EP	0.391
AIESMA and ICC	0.594
IIE and CII	0.525
IIE and EP	0.468
IIE and ICC	0.714
CII and EP	0.426
CII and ICC	0.698
EP and ICC	0.487

HTMT was used as an additional diagnostic for discriminant validity. All values were below 0.85. Fornell-Larcker discriminant-validity evidence is reported in Supplementary File 6.

The reflective-formative higher-order AIESMA construct was then assessed using the two-stage approach. The five lower-order affordance dimensions were first estimated reflectively, and their latent variable scores were then used as indicators of the higher-order formative AIESMA construct. As shown in Table 8, all five lower-order dimensions had significant weights and acceptable VIF values. AI-supported cultural interpretation had the largest weight, followed by AI-enabled pragmatic feedback, algorithmic cultural exposure, AI-assisted expression support, and AI-mediated comprehension support. This pattern suggests that culturally embedded meaning-making and pragmatic interpretation were especially central to how Chinese business students perceived AI-enhanced social media affordances.

**TABLE 8:** Reflective-formative higher-order AIESMA assessment.

Lower-order dimension	Weight	Loading	VIF	*t*	*P*
Algorithmic cultural exposure	0.244	0.789	1.914	5.545	< 0.001
AI-mediated comprehension support	0.217	0.764	1.743	5.047	< 0.001
AI-assisted expression support	0.232	0.781	1.866	5.273	< 0.001
AI-enabled pragmatic feedback	0.254	0.806	2.096	5.907	< 0.001
AI-supported cultural interpretation	0.286	0.842	2.004	6.5	< 0.001

AIESMA was modeled as a reflective-formative higher-order construct. Lower-order dimensions were measured reflectively, while the higher-order AIESMA construct was modeled formatively.

Potential common method variance was also assessed. Harman’s single-factor test showed that the first factor accounted for 31.42% of the total variance, and the maximum full collinearity VIF was 2.71. These diagnostics did not indicate a dominant single-factor pattern, but they cannot rule out common method variance. Detailed common method variance diagnostics are reported in Supplementary File 7.

### 4.4 Structural model and direct associations

After the measurement model showed acceptable reliability and validity, the structural model was assessed. Table 9 reports the structural model quality indicators for the final structural specification. The maximum inner VIF values were below the recommended threshold, indicating that multicollinearity was not a serious concern. The model explained 43.6% of the variance in intercultural interaction engagement (IIE), 19.7% of the variance in cultural identity integration (CII), and 43.8% of the variance in intercultural communication competence (ICC). These values indicated meaningful explanatory power for IIE and ICC and more modest explanatory power for CII. The Q^2^_predict values also suggested predictive relevance for the endogenous constructs. In addition, the SRMR value was 0.049, indicating an acceptable model diagnostic for the PLS-SEM analysis.

**TABLE 9:** Structural model quality indicators.

Endogenous construct	*R*-square	Adjusted *R*-square	*Q*^2^_predict	Maximum inner VIF	Predictive interpretation
IIE	0.436	0.434	0.315	1.374	Medium
CII	0.197	0.195	0.142	1.567	Low to medium
ICC	0.438	0.436	0.306	2.014	Medium
Model diagnostic	Not applicable	Not applicable	Not applicable	Not applicable	SRMR = 0.049

Q^2^_predict values summarize predictive relevance. SRMR is reported as a model diagnostic rather than as a covariance-based fit index. For IIE, R-square and inner VIF values were calculated from the moderation specification including AIESMA, EP, and the AIESMA × EP interaction term.

Table 10 reports the direct path estimates. Five of the six direct hypotheses were statistically supported. AIESMA was positively associated with IIE, supporting H1. AIESMA was also positively associated with CII, supporting H2, although the size of this direct association was relatively small. IIE was positively associated with CII, supporting H3. IIE and CII were both positively associated with self-reported ICC, supporting H4 and H5. However, the direct association from AIESMA to self-reported ICC was not statistically significant after IIE and CII were included in the model. Therefore, H6 was not supported. This result should not be interpreted as evidence of no relationship between AIESMA and ICC. Instead, the AIESMA–ICC relationship was primarily reflected in engagement-centered indirect associations.

**TABLE 10:** Direct path coefficients and hypothesis tests.

Hypothesis	Path	Beta	SE	*t*	*p*	95% CI	Decision
H1	AIESMA to IIE	0.518	0.035	14.8	< 0.001	[0.450, 0.586]	Supported
H2	AIESMA to CII	0.092	0.041	2.244	0.025	[0.012, 0.173]	Supported
H3	IIE to CII	0.382	0.039	9.795	< 0.001	[0.306, 0.459]	Supported
H4	IIE to ICC	0.321	0.041	7.829	< 0.001	[0.241, 0.402]	Supported
H5	CII to ICC	0.348	0.04	8.7	< 0.001	[0.270, 0.426]	Supported
H6	AIESMA to ICC	0.061	0.038	1.605	0.109	[−0.014, 0.136]	Not supported

Bootstrapping used 5,000 resamples. Results show associations in a survey-based PLS-SEM model and should not be interpreted as causal estimates.

### 4.5 Mediation, moderation, and moderated mediation

Table 11 reports the mediation and sequential mediation results. The indirect association between AIESMA and self-reported ICC through IIE was significant, supporting H7. However, the indirect association through CII alone was not significant; therefore, H8 was not supported. The theoretically specified sequential indirect association through IIE and CII was significant, supporting H9. In this statistical ordering, IIE was modeled before CII based on the theoretical specification; the cross-sectional data do not establish temporal order. These results did not provide sufficient statistical evidence that CII independently mediated the AIESMA–ICC association. CII nevertheless had a statistically significant direct association with self-reported ICC, and its indirect explanatory role was observed within the model-specified sequential pathway through IIE and CII.

**TABLE 11:** Mediation and sequential mediation results.

Hypothesis	Indirect path	Effect	SE	*t*	*P*	95% CI	Decision
H7	AIESMA to IIE to ICC	0.166	0.025	6.64	< 0.001	[0.118, 0.217]	Supported
H8	AIESMA to CII to ICC	0.032	0.019	1.684	0.092	[−0.005, 0.073]	Not supported
H9	AIESMA to IIE to CII to ICC	0.069	0.015	4.6	< 0.001	[0.042, 0.102]	Supported
Not hypothesized	Total indirect association	0.267	0.039	6.846	< 0.001	[0.193, 0.346]	Significant
Not hypothesized	Total association	0.328	0.043	7.628	< 0.001	[0.244, 0.413]	Significant

The table reports indirect associations rather than causal mediation effects. Bootstrapping used 5,000 resamples.

Among the indirect associations, the AIESMA-to-IIE-to-ICC association had the largest specific indirect estimate. Intercultural interaction engagement therefore served as the main indirect explanatory path linking AIESMA with self-reported ICC. The indirect association through CII alone was not statistically significant, so the evidence did not support an independent mediating role for CII in the AIESMA–ICC association. The theoretically specified sequential indirect association through IIE and CII was significant. Thus, the indirect explanatory role of CII was observed within the model-specified statistical ordering that included IIE. Because the direct AIESMA–ICC path was not statistically significant, but the indirect associations through IIE and through IIE and CII were significant, the overall pattern supports an engagement-centered indirect association rather than a direct affordance-to-competence association.

Table 12 shows the moderation test and simple-slope results. The interaction between AIESMA and self-reported EP was significant, supporting H10. The simple slopes showed that the association between AIESMA and IIE was significant at low, mean, and high levels of EP, and the association was strongest among higher-proficiency students. This pattern indicates that higher self-reported EP was associated with a stronger AIESMA–IIE relationship in this sample.

**TABLE 12:** English proficiency moderation and simple-slope results.

Test	Estimate	SE	*t*	*P*	95% CI	Interpretation
AIESMA → IIE	0.518	0.035	14.800	<0.001	[0.450, 0.586]	Main effect (H1)
EP → IIE	0.231	0.033	7.000	<0.001	[0.166, 0.296]	Non-hypothesized main effect
AIESMA × EP → IIE	0.105	0.033	3.182	0.001	[0.041, 0.170]	H10 supported
Low EP simple slope	0.416	0.044	9.455	<0.001	[0.331, 0.503]	Significant
Mean EP simple slope	0.518	0.035	14.8	<0.001	[0.450, 0.586]	Significant
High EP simple slope	0.62	0.047	13.191	<0.001	[0.529, 0.714]	Stronger

Low and high EP represent one standard deviation below and above the mean. The EP → IIE row is a non-hypothesized main effect and has no hypothesis number.

Table 13 reports the moderated mediation results. Two conditional indirect patterns were examined. First, the conditional indirect association through IIE alone was significant and stronger at high EP than at low EP. This result was reported as an additional conditional indirect association because it corresponded to the first-stage moderation of the AIESMA-to-IIE path. Second, the conditional sequential indirect association through IIE and CII was significant. This second pathway directly corresponded to H11, which proposed that English proficiency moderates the theoretically specified sequential indirect association between AIESMA and ICC through IIE and CII.

**TABLE 13:** Moderated mediation results.

Analysis	Effect or index	SE	Bootstrapped 95% CI	Interpretation
Index: AIESMA to IIE to ICC	0.034	0.012	[0.011, 0.059]	Additional conditional indirect association
Index: AIESMA to IIE to CII to ICC	0.014	0.006	[0.004, 0.028]	H11 supported
Low EP: AIESMA to IIE to ICC	0.134	0.026	[0.087, 0.189]	Significant
High EP: AIESMA to IIE to ICC	0.199	0.033	[0.139, 0.269]	Stronger
Low EP: AIESMA to IIE to CII to ICC	0.055	0.013	[0.032, 0.084]	Significant
High EP: AIESMA to IIE to CII to ICC	0.083	0.018	[0.050, 0.123]	Stronger

The table reports conditional indirect associations rather than causal moderated mediation effects. Confidence intervals were estimated using bootstrapping with 5,000 resamples.

Overall, the mediation, moderation, and moderated mediation results provided partial support for the proposed theoretical model. AIESMA did not show a statistically significant direct association with self-reported ICC after IIE and CII were included, but it showed significant indirect associations through IIE and through the theoretically specified sequential pathway through IIE and CII. The unsupported H8 result indicates that the evidence was insufficient for an independent mediating role of CII in the AIESMA–ICC association. CII was directly associated with self-reported ICC, but its indirect explanatory role was limited to the model-specified sequential pathway through IIE and CII. EP functioned as a boundary condition associated with differences in the AIESMA–IIE association and the conditional sequential indirect association. Although the moderated mediation index was statistically significant, its magnitude was modest, suggesting that English proficiency was a meaningful but not dominant boundary condition. Robustness checks using platform-exposure grouping and alternative construct specifications produced substantively consistent results, as reported in Supplementary File 8. Alternative model comparisons supported the plausibility of the theoretically specified statistical ordering but did not provide evidence of temporal sequence, as reported in Supplementary File 9. A supplementary test-based relative proficiency robustness check was conducted for 476 respondents with a valid reported score from CET-4, CET-6, IELTS, TOEFL, or BEC. Each respondent contributed one primary test indicator; when multiple valid scores were available, the score closest in time to the survey was selected. Scores were standardized within each test type using *z* = (individual score − mean for that test)/standard deviation for that test, and test type indicators were included as controls. The resulting z-scores represented relative standing within test type, not absolute equivalence across tests and not a superior substitute for the main self-reported EP measure. The supplementary interaction estimate was β = 0.087, SE = 0.039, *t* = 2.231, *p* = 0.026, 95% CI [0.011, 0.163]. This directionally consistent result did not replace the main moderation estimate. Detailed procedures are reported in Supplementary File 10.

## 5 Discussion

### 5.1 Summary and interpretation of findings

This study examined how AI-enhanced social media affordances were associated with self-reported intercultural communication competence (ICC) among Chinese business students through intercultural interaction engagement and cultural identity integration, with self-reported English proficiency as a boundary condition. Overall, the findings provided partial support for the proposed model. AIESMA was positively associated with intercultural interaction engagement and cultural identity integration, but its direct association with self-reported ICC was not statistically significant after intercultural interaction engagement and cultural identity integration were included. Intercultural interaction engagement and cultural identity integration were both positively associated with self-reported ICC. The indirect association through intercultural interaction engagement and the theoretically specified sequential indirect association through intercultural interaction engagement and cultural identity integration were significant, whereas the indirect association through cultural identity integration alone was not statistically significant. The moderation and moderated mediation results were also statistically significant. Because the main model used cross-sectional survey data, these results should be interpreted as theoretically specified statistical associations rather than causal effects or evidence of temporal sequence.

Among the direct associations, the strongest relationship was between AIESMA and intercultural interaction engagement. This finding indicates that perceived AI-supported platform functions were most closely associated with students’ self-reported engagement with intercultural content, comments, discussions, and communities. Recent EFL research similarly shows that perceived AI affordances are closely associated with students’ behavioral, cognitive, emotional, and social engagement (Xu and Li, 2024). In this study, recommendation, translation, subtitles, AI-assisted writing, pragmatic feedback, and cultural explanation were not simply technical tools; they were associated with students’ opportunities to observe, interpret, reply, compare, and reflect in business-related social media environments. This interpretation is consistent with recent social media research showing that engagement features acquire communicative and relational value through the interaction between technological infrastructures, social contexts, and user practices (Scharlach and Hallinan, 2023).

The direct association between AIESMA and self-reported ICC was not statistically significant after intercultural interaction engagement and cultural identity integration were included. This non-significant result is theoretically meaningful because it suggests that AI-enhanced affordances should not be treated as a direct substitute for intercultural learning. Access to AI translation, subtitles, recommendation, or writing support is not by itself evidence of higher self-reported ICC. Research on mobile language learning similarly indicates that technological affordances acquire educational relevance through learner actualization in specific activities (Tong et al., 2024). In the present model, AIESMA was associated with self-reported ICC primarily through engagement-centered indirect associations involving culturally diverse meanings, perspective comparison, expression, and platform-based communication. A recent systematic review of AI-supported English learning also concludes that AI functions are most beneficial when embedded in purposeful language use, learner engagement, and critical evaluation rather than passive technological adoption (Crompton et al., 2024).

This interpretation is further supported by the fact that generative AI may offer more access to feedback and personalized language support, but at the same time, it can produce culturally simplified or contextually inappropriate language (Lo, 2025). Therefore, students need to evaluate AI-generated explanations and expressions rather than passively accept them. A recent scoping review has also found that effective applications of generative AI in language learning require informed use, pedagogical objectives, and active learner participation (Law, 2024).

Cultural identity integration showed a slightly stronger association with self-reported ICC than intercultural interaction engagement among the direct predictors of ICC. This result suggests that perceived business-relevant ICC was associated not only with reported interaction and digital participation, but also with how students positioned themselves culturally in global communication. Students who reported connecting Chinese cultural identity with global business participation also reported greater ability to explain Chinese perspectives, respond to difference without defensiveness, and adapt communication strategies without losing cultural self-understanding. Recent organizational research similarly shows that individuals with more integrated cultural identities possess stronger cultural and linguistic bridging capabilities than those whose cultural identities remain separated or ambiguous (Popescu and Pudelko, 2024). Research on multilingual university students further indicates that digital participation can become an important setting in which learners negotiate linguistic, cultural, and social identities (Ye, 2025). However, cultural identity integration did not independently mediate the AIESMA–ICC association; its indirect explanatory role was observed only within the model-specified sequential association through IIE and CII.

The mediation results further clarified the relative importance of engagement and identity integration. The indirect association through intercultural interaction engagement was significant and represented the largest specific indirect association. This indicates that engagement was the most salient explanatory link between AIESMA and self-reported ICC in this sample. Recent virtual-exchange research similarly links intercultural development with learners’ engagement with communication partners, cultural differences, and interactional challenges (Rets et al., 2023). Evidence from informal digital English learning also shows that receptive exposure and productive participation in online English activities are positively associated with intercultural competence among Chinese university students (Liu et al., 2025).

The indirect association through cultural identity integration alone was not statistically significant; therefore, the data did not support an independent mediating role for CII in the AIESMA–ICC association. The theoretically specified sequential indirect association through intercultural interaction engagement and cultural identity integration was significant. The theoretical model placed intercultural interaction engagement before cultural identity integration for hypothesis testing, but the cross-sectional data do not demonstrate a real-time temporal sequence. This statistical pattern is consistent with research in Chinese business English education linking intercultural development with noticing, comparison, interaction, and reflection (Mu and Yu, 2023).

Higher self-reported English proficiency was associated with a stronger AIESMA–IIE relationship in this sample. The simple slopes were significant at low, mean, and high levels of English proficiency, and the association was strongest for higher-proficiency students. Higher-proficiency students may have been better able to evaluate AI outputs, recognize pragmatic differences, correct AI-assisted expressions, and engage in extended intercultural communication. Recent experimental evidence has shown that conversational generative AI can increase learners’ willingness to communicate and self-assessed communicative competence, although this does not automatically lead to improvements in actual language performance (Wang et al., 2024).

The association between AIESMA and IIE remained statistically significant at lower levels of self-reported English proficiency, although it was weaker than the corresponding association at higher proficiency levels. AI-assisted functions may therefore still offer support to students with weaker English proficiency. Generative AI tools can provide translation, explanation, language modeling, and revision support, while effective pedagogical use still requires critical AI literacy (Kohnke et al., 2023; Crompton et al., 2024). Higher-proficiency learners may nevertheless be better able to verify AI output, recognize inappropriate expressions, and use AI support for more advanced communication. Research on informal digital English learning also indicates that students with stronger linguistic and psychological resources engage more deeply in digital learning and communicate with greater intercultural sensitivity (Liu and Lee, 2026).

Unlike prior studies that examined social media as a general channel for intercultural communication between university students, this study focuses on AI-enhanced social media affordances and explains how AI-supported recommendation, translation, subtitles, writing assistance, and cultural explanation are associated with business-relevant ICC among Chinese business students. It also differs from recent reviews of ICC in EFL education and global business by providing a two-study empirical model that integrates intercultural engagement, cultural identity integration, and English proficiency (Xu et al., 2025). Therefore, the study shifts the focus from whether social media supports intercultural communication to how AI-enhanced platform affordances become associated with ICC mainly through engagement-centered and sequential identity-related processes.

### 5.2 Theoretical contributions

This study makes four theoretical contributions. First, it extends affordance theory by specifying how AI-enhanced social media affordances are associated with self-reported intercultural communication competence in business education. A recent systematic review indicates that the pedagogical uses of generative AI vary across learners, educators, purposes, and contexts (Qian, 2025). From this relational view, AIESMA is not limited to technical functions such as algorithmic recommendations, machine translation, automatic subtitles, AI-assisted writing, or AI-based explanations. Its theoretical value lies in showing how students perceive these functions as opportunities for intercultural contact, understanding, expression, feedback, and cultural exploration. The nonsignificant direct AIESMA–ICC association is also consistent with the view that digital affordances should not be equated with communicative competence; their value is associated with user actualization through intercultural engagement. Social media research likewise indicates that platform affordances acquire communicative value through users’ interpretation and practice in context (Scharlach and Hallinan, 2023).

Second, the study adds to research on intercultural communication competence by examining ICC in AI-assisted, English-mediated, and business-oriented social media environments. Research on intercultural communication often views communicative competence as the negotiation of language and culture in situated interaction rather than the inheritance of a fixed national-cultural system (Baker, 2024). This paper extends that work by showing that Chinese business students’ self-reported ICC was associated with platform-based participation, pragmatic judgment, multimodal cue interpretation, and culturally rooted self-positioning. English proficiency did not substitute for ICC; it was associated with differences in how students actualized the intercultural potential of AIESMA. Research also identifies digital communication, virtual exchange, and online interaction as increasingly important contexts for intercultural communication in second- and foreign-language education (Cong-Lem, 2025).

This contribution is especially relevant to business education because interactional effectiveness must be enacted in consumer discourse, brand narratives, livestreams, service complaints, professional discussions, and other context-dependent communication settings. Research on AI-supported professional communication similarly emphasizes that culturally appropriate workplace communication requires contextual judgment and interactional competence that cannot be reduced to grammatically accurate AI-generated language (Dai et al., 2025). The present study therefore extends ICC from predominantly classroom-based contexts to everyday platform-based business communication.

Third, the study advances theory by clarifying the different roles of intercultural engagement and cultural identity integration in AI-enhanced intercultural learning. Recent virtual-exchange research links intercultural competence with sustained interaction, engagement with difference, negotiation, and reflection rather than digital contact alone (Rets et al., 2023). The present results indicate that intercultural interaction engagement was the strongest indirect explanatory link between AIESMA and self-reported ICC. Research on informal digital English learning similarly associates active online participation with learners’ linguistic and psychological resources and intercultural competence (Liu and Lee, 2026).

Cultural identity integration was directly associated with self-reported ICC, indicating that perceived intercultural competence was not only interactional but also identity-oriented. Recent research shows that an integrated cultural identity strengthens individuals’ capacity to operate as cultural and linguistic bridges across different communication environments (Popescu and Pudelko, 2024). However, cultural identity integration did not independently mediate the AIESMA–ICC association; its indirect explanatory role was observed within the theoretically specified sequential association through IIE and CII. This cross-sectional association is consistent with, but does not establish, the theorized ordering in which engagement is modeled before identity integration. Longitudinal research on technology-mediated identity negotiation indicates that cultural and linguistic identities are reconstructed through repeated digital participation and social positioning (Ye, 2025).

Fourth, English proficiency functions as a boundary condition associated with differences in affordance actualization rather than as a substitute for intercultural communication competence. Research on virtual exchange indicates that learners’ language ability is related to access, confidence, and participation in online intercultural communication, while technological access alone does not resolve linguistic challenges (O’Dowd, 2025). In the present study, self-reported English proficiency was associated with differences in the AIESMA–IIE relationship. Higher-proficiency students may be better able to use translation, subtitles, and AI-assisted writing to examine differences in expression and revise their communication, while lower-proficiency students could also use these functions as support.

This difference is also relevant to research on generative AI in language education. AI tools can extend access to language input, feedback, and communication practice; however, learners still need linguistic knowledge to recognize inaccurate, culturally insensitive, or pragmatically inappropriate output (Kohnke et al., 2023; Crompton et al., 2024). Accordingly, the findings should not be interpreted as evidence that AI enhances learning in general. Instead, its theoretical significance lies in the observed engagement-centered associations and in the model-specified roles of cultural identity integration and learner language resources.

### 5.3 Practical implications

Instructors can ask students to analyze foreign consumer comments, compare how brands respond to complaints, and participate in moderated English-mediated discussions. AI translation and subtitles should not be used merely for quick understanding; students need to compare the original and translated meanings, identify pragmatic risks, and revise the AI-assisted replies.

Curriculum design should link language, culture, business discourse and identity in ICC training. Business English, cross-border e-commerce, international marketing, tourism communication and digital trade courses can integrate platform-based activities such as user-generated content, comments, live streams and community norms. Reflection activities should have students present Chinese business practices to an international audience and adapt their communication to the relevant context without losing cultural connotations. Given that the independent indirect association through CII was not statistically significant, the findings suggest that identity reflection may be more appropriately integrated with intercultural interaction tasks rather than treated as sufficient on its own.

Designers of social media platforms and educational technologies should develop AI tools that support culturally appropriate participation rather than literal translation alone. Platforms and learning systems can explain pragmatic tones, politeness, humor, consumer emotions, and culturally sensitive language, and support learners in formulating contextually appropriate responses. Adaptable support is required to provide a learning scaffold for less proficient learners and, at the same time, offer rich pragmatic feedback and more advanced language refinement for more proficient learners.

### 5.4 Limitations and future research

Some limitations remain. First, although the model was theoretically specified and tested in a two-study design, the cross-sectional data do not establish causality or temporal order. Future research could employ longitudinal designs, experiments, classroom interventions, or learning analytics to examine whether AI-assisted engagement and identity integration change over time. Second, all five main constructs were reported by the same participant at a single time point in the same questionnaire. The common-method diagnostics did not indicate a dominant single-factor pattern, but they cannot rule out common method variance. Most variables, including ICC, were self-reported, so the ICC scores represent perceived competence rather than demonstrated performance. Future work could include performance-based ICC evaluations, discourse analysis of actual comments and replies, peer evaluations, instructor ratings, scenario-based business communication tasks, or platform-based behavioral data. Each IIE dimension was represented by two items; future research could test broader item coverage while preserving the four-dimensional structure. Third, the sample included only Chinese business students. Although this focus increased contextual relevance, it limits generalizability to other national, disciplinary, and professional groups. Future work could compare these results across countries, professionals, language majors, engineering students, and vocational students. Fourth, platform ecologies differ in algorithmic transparency, moderation norms, audience expectations, multimodal formats, and AI support. Future research could investigate platform-specific affordances rather than assuming a single model of social media. Fifth, AI tools are changing rapidly. Future research could examine emerging affordances and risks, including overreliance, cultural homogeneity, pragmatic misalignment, algorithmic bias, privacy concerns, and unequal access to high-quality AI support. Sixth, the business-relevant ICC scale requires further cross-validation across cultures, majors, platforms, and proficiency groups, ideally with behavioral or performance-based indicators. Finally, future research could examine why CII’s independent indirect association was not statistically significant, whereas the model-specified sequential indirect association involving IIE and CII was significant.

## 6 Conclusion

The study validated a multidimensional measurement model and provided partial support for the proposed model linking AIESMA, IIE, CII, and ICC. AIESMA did not show a statistically significant direct association with self-reported ICC after IIE and CII were included, and CII did not independently mediate the AIESMA–ICC association. The findings indicate that AIESMA was associated with self-reported ICC primarily through engagement-centered indirect associations and the theoretically specified sequential association through IIE and CII. These results do not imply that AI-enhanced social media is an automatic source of intercultural communication competence.

Cultural identity integration was directly associated with self-reported ICC and formed part of the significant theoretically specified sequential indirect association through IIE and CII. Stronger cultural identity integration was associated with higher self-reported ICC, including greater perceived ability to connect Chinese cultural grounding with global business communication. The findings were consistent with a theoretically specified pathway in which IIE was positioned before CII, but the cross-sectional data do not establish actual temporal order. Self-reported English proficiency functioned as a modest boundary condition: the AIESMA–IIE association was significant at all proficiency levels but stronger at higher proficiency. In short, platform-based AI was associated with self-reported ICC primarily through engagement-centered indirect associations and the model-specified engagement-to-identity pathway, rather than directly through technological exposure. AI-enhanced social media may serve as a structured environment for intercultural observation, participation, reflection, identity negotiation, and professional communication practice in Chinese business education.

## Data Availability

The original contributions presented in the study are included in the article/Supplementary material, further inquiries can be directed to the corresponding author.
