# Supplementary material for: AI-enhanced social media affordances and intercultural communication competence: engagement, identity integration, and English proficiency
Source: Front Psychol. 2026 Sep 14;17:1960943. doi: 10.3389/fpsyg.2026.1960943 (PMC13617104; doi:10.3389/fpsyg.2026.1960943)
Supplement: Supplementary file 1 [file Table_1.docx]

# Supplementary Materials: Files 1–10

Manuscript: AI-Enhanced Social Media Affordances and Intercultural Communication Competence: Engagement, Identity Integration, and English Proficiency

Combined submission file

This document consolidates Supplementary Files 1–10 while retaining the numbering used in the main manuscript.

## Organization and data map

File 1 — Measurement items and retention: 57 initial items; 51 retained (AIESMA 15, IIE 8, CII 6, EP 5, ICC 17).

File 2 — Expert review and refinement: 8 experts; I-CVI decision rule ≥ .78; 24 cognitive-interview participants; 6 deletions (3 AIESMA, 2 IIE, 1 CII).

File 3 — Study 1 EFA: principal axis factoring with Promax oblique rotation; five AIESMA factors, four IIE factors, six ICC factors, and one factor each for CII and EP; 51 items retained.

File 4 — Study 2 standardized loadings: 51 retained items.

File 5 — Additional discriminant-validity summary: HTMT and Fornell-Larcker evidence.

File 6 — Fornell-Larcker evidence: construct correlations and applicable square roots of AVE.

File 7 — Common method variance diagnostics: first Harman factor 31.42%; maximum full collinearity VIF 2.71; residual common method variance cannot be ruled out.

File 8 — Robustness checks: platform-exposure grouping and alternative construct specifications.

File 9 — Alternative model comparisons: structural sensitivity analyses.

File 10 — Test-based relative EP check: N = 476; CET-4/CET-6/IELTS/TOEFL/BEC; β = .087, SE = .039, t = 2.231, p = .026, 95% CI [.011, .163].

# Supplementary File 1. Full measurement items and item-retention decisions

Manuscript: AI-Enhanced Social Media Affordances and Intercultural Communication Competence: Engagement, Identity Integration, and English Proficiency

This section provides the complete 57-item initial pool and the 51-item retained instrument. AIESMA items were newly developed; IIE, EP, and ICC items were contextually developed; CII items were adapted and contextualized. Final retained counts were AIESMA = 15, IIE = 8, CII = 6, EP = 5, and ICC = 17.

## Supplementary Table S1. Initial item pool and item-retention decisions

Note. Six items were removed after expert review and cognitive interviews before the final Study 1 EFA: three AIESMA items, two IIE items, and one CII item. The remaining 51 items formed the Study 2 questionnaire.

| **Construct** | **Dimension** | **Item code** | **Item wording** | **Decision** | **Reason for deletion if applicable** |
| --- | --- | --- | --- | --- | --- |
| AIESMA | Algorithmic cultural exposure | ACE1 | AI-enhanced social media exposes me to culturally diverse business content. | Retained | Not applicable |
|  |  | ACE2 | Platform recommendations help me encounter international consumer opinions. | Retained | Not applicable |
|  |  | ACE3 | AI-curated feeds broaden my awareness of cross-cultural market practices. | Retained | Not applicable |
|  |  | ACE4 | AI recommendations help me understand unfamiliar cultural content. | Deleted | Deleted after expert review because of conceptual overlap |
|  | AI-mediated comprehension support | MCS1 | AI translation helps me understand foreign-language business posts. | Retained | Not applicable |
|  |  | MCS2 | Automatic subtitles help me follow intercultural business videos. | Retained | Not applicable |
|  |  | MCS3 | AI explanations help me understand culturally specific online meanings. | Retained | Not applicable |
|  |  | MCS4 | AI tools reduce uncertainty in cross-cultural communication. | Deleted | Deleted after cognitive interviews because of ambiguous wording |
|  | AI-assisted expression support | AES1 | AI writing support helps me express ideas in intercultural online exchanges. | Retained | Not applicable |
|  |  | AES2 | AI suggestions help me choose appropriate wording for international audiences. | Retained | Not applicable |
|  |  | AES3 | AI assistance increases my confidence in business-related online expression. | Retained | Not applicable |
|  | AI-enabled pragmatic feedback | APF1 | AI feedback helps me notice tone problems in intercultural messages. | Retained | Not applicable |
|  |  | APF2 | AI suggestions help me adjust politeness in online business communication. | Retained | Not applicable |
|  |  | APF3 | AI feedback helps me improve pragmatic appropriateness in social media replies. | Retained | Not applicable |
|  |  | APF4 | AI feedback mainly helps me improve general grammar accuracy. | Deleted | Deleted because of weak business-context fit |
|  | AI-supported cultural interpretation | SCI1 | AI explanations help me interpret cultural references in social media content. | Retained | Not applicable |
|  |  | SCI2 | AI tools help me compare different cultural perspectives in online discussions. | Retained | Not applicable |
|  |  | SCI3 | AI support helps me understand values behind international consumer discourse. | Retained | Not applicable |
| IIE | Content engagement | CE1 | I actively read intercultural business content on social media. | Retained | Not applicable |
|  |  | CE2 | I pay attention to cultural meanings in international online content. | Retained | Not applicable |
|  |  | CE3 | I often think about online intercultural content after viewing it. | Deleted | Deleted because of overlap with reflective engagement |
|  | Interactive engagement | IE1 | I comment or reply in intercultural social media discussions. | Retained | Not applicable |
|  |  | IE2 | I ask questions when interacting with people from other cultures online. | Retained | Not applicable |
|  | Reflective engagement | RE1 | I reflect on cultural differences after online intercultural interactions. | Retained | Not applicable |
|  |  | RE2 | I compare my assumptions with perspectives encountered on social media. | Retained | Not applicable |
|  |  | RE3 | I usually remember intercultural posts that I find interesting. | Deleted | Deleted because of unclear wording in cognitive interviews |
|  | Relational engagement | RLE1 | I maintain online connections with people from different cultural backgrounds. | Retained | Not applicable |
|  |  | RLE2 | I use social media to build relationships across cultural groups. | Retained | Not applicable |
| CII | Cultural identity integration | CII1 | I can integrate Chinese cultural identity with global business perspectives. | Retained | Not applicable |
|  |  | CII2 | Intercultural contact helps me understand my own cultural identity more clearly. | Retained | Not applicable |
|  |  | CII3 | I can respect other cultures while maintaining my cultural background. | Retained | Not applicable |
|  |  | CII4 | I feel comfortable combining local and international viewpoints. | Retained | Not applicable |
|  |  | CII5 | I can explain Chinese cultural perspectives in intercultural contexts. | Retained | Not applicable |
|  |  | CII6 | I see cultural identity as flexible during intercultural communication. | Retained | Not applicable |
|  |  | CII7 | I feel proud of my culture in a general sense. | Deleted | Deleted because of overlap with general cultural pride |
| EP | English proficiency | EP1 | I can understand English posts related to business or marketing. | Retained | Not applicable |
|  |  | EP2 | I can write English comments for professional online communication. | Retained | Not applicable |
|  |  | EP3 | I can follow English videos about international business topics. | Retained | Not applicable |
|  |  | EP4 | I can express opinions in English during intercultural online discussions. | Retained | Not applicable |
|  |  | EP5 | I can use English to solve communication problems on social media. | Retained | Not applicable |
| ICC | Cultural understanding | CU1 | I understand cultural values reflected in intercultural business communication. | Retained | Not applicable |
|  |  | CU2 | I can recognize culturally specific meanings in online messages. | Retained | Not applicable |
|  |  | CU3 | I understand how business communication norms vary across cultures. | Retained | Not applicable |
|  | Intercultural sensitivity | IS1 | I show respect when communicating with people from other cultures. | Retained | Not applicable |
|  |  | IS2 | I am open to unfamiliar cultural viewpoints during online interaction. | Retained | Not applicable |
|  |  | IS3 | I avoid judging intercultural messages only by my own cultural norms. | Retained | Not applicable |
|  | Communication strategies | CS1 | I use strategies to clarify misunderstandings in intercultural communication. | Retained | Not applicable |
|  |  | CS2 | I adjust my communication style for different cultural audiences. | Retained | Not applicable |
|  |  | CS3 | I ask for clarification when cultural meanings are unclear. | Retained | Not applicable |
|  | Pragmatic appropriateness | PA1 | I choose appropriate levels of politeness in intercultural exchanges. | Retained | Not applicable |
|  |  | PA2 | I adapt tone and wording to fit intercultural business contexts. | Retained | Not applicable |
|  |  | PA3 | I can respond appropriately to culturally sensitive online situations. | Retained | Not applicable |
|  | Multimodal cue interpretation | MCI1 | I can interpret visual and verbal cues in intercultural social media content. | Retained | Not applicable |
|  |  | MCI2 | I notice how images, emojis, and tone affect intercultural meaning. | Retained | Not applicable |
|  | Interactional effectiveness | IEF1 | I can achieve communication goals in intercultural online interactions. | Retained | Not applicable |
|  |  | IEF2 | I can maintain effective interaction when cultural differences arise. | Retained | Not applicable |
|  |  | IEF3 | I can participate successfully in business-related intercultural communication. | Retained | Not applicable |

**Supplementary File 2. Expert review and item refinement**

Manuscript: AI-Enhanced Social Media Affordances and Intercultural Communication Competence: Engagement, Identity Integration, and English Proficiency

This file documents how expert review and cognitive interviews were used to refine the item pool before Study 1 EFA.

## Procedure summary

The initial item pool contained 57 items: 18 AIESMA items, 10 IIE items, 7 CII items, 5 EP items, and 17 ICC items. Eight experts—two applied linguistics scholars, two intercultural communication scholars, two business English or international business communication instructors, and two educational technology researchers—rated item relevance on a four-point scale and evaluated conceptual fit, clarity, contextual suitability, redundancy, and construct representation. An item-level content validity index (I-CVI) of at least .78 was the quantitative retention rule unless qualitative comments identified a substantive conceptual or wording problem. Cognitive interviews with 24 Chinese business students then examined comprehension and contextual fit. Six items were removed: three AIESMA items, two IIE items, and one CII item. No EP or ICC items were removed.

## Supplementary Table S2. Deleted items after expert review and cognitive interviews

| Construct | Dimension | Item code | Item wording | Decision | Reason for deletion if applicable |
| --- | --- | --- | --- | --- | --- |
| AIESMA | Algorithmic cultural exposure | ACE4 | AI recommendations help me understand unfamiliar cultural content. | Deleted | Deleted after expert review because of conceptual overlap |
| AIESMA | AI-mediated comprehension support | MCS4 | AI tools reduce uncertainty in cross-cultural communication. | Deleted | Deleted after cognitive interviews because of ambiguous wording |
| AIESMA | AI-enabled pragmatic feedback | APF4 | AI feedback mainly helps me improve general grammar accuracy. | Deleted | Deleted because of weak business-context fit |
| IIE | Content engagement | CE3 | I often think about online intercultural content after viewing it. | Deleted | Deleted because of overlap with reflective engagement |
| IIE | Reflective engagement | RE3 | I usually remember intercultural posts that I find interesting. | Deleted | Deleted because of unclear wording in cognitive interviews |
| CII | Cultural identity integration | CII7 | I feel proud of my culture in a general sense. | Deleted | Deleted because of overlap with general cultural pride |

Note. Individual expert-rating scores are not presented in this supplement.

# Supplementary File 3. Study 1 exploratory factor analysis loadings

Manuscript: AI-Enhanced Social Media Affordances and Intercultural Communication Competence: Engagement, Identity Integration, and English Proficiency

This section reports primary pattern-matrix loadings for all 51 retained Study 1 items. EFA used principal axis factoring with Promax oblique rotation. Parallel analysis and scree plot inspection supported five factors for AIESMA, four factors for IIE, six factors for ICC, and one factor each for CII and EP.

## Supplementary Table S3. Study 1 retained items and EFA loadings

Note. All 51 items were retained. Criteria were primary loading ≥ .50, communality ≥ .40, and theoretical interpretability; for multidimensional constructs, cross-loadings < .30 and a primary-to-secondary difference ≥ .20. Table S3 reports primary pattern-matrix loadings for the retained items.

| **Construct** | **Dimension** | **Item code** | **Primary pattern-matrix loading** |
| --- | --- | --- | --- |
| AIESMA | Algorithmic cultural exposure | ACE1 | 0.742 |
|  |  | ACE2 | 0.831 |
|  |  | ACE3 | 0.884 |
|  | AI-mediated comprehension support | MCS1 | 0.761 |
|  |  | MCS2 | 0.842 |
|  |  | MCS3 | 0.895 |
|  | AI-assisted expression support | AES1 | 0.778 |
|  |  | AES2 | 0.856 |
|  |  | AES3 | 0.901 |
|  | AI-enabled pragmatic feedback | APF1 | 0.753 |
|  |  | APF2 | 0.871 |
|  |  | APF3 | 0.914 |
|  | AI-supported cultural interpretation | SCI1 | 0.792 |
|  |  | SCI2 | 0.868 |
|  |  | SCI3 | 0.907 |
| IIE | Content engagement | CE1 | 0.758 |
|  |  | CE2 | 0.884 |
|  | Interactive engagement | IE1 | 0.791 |
|  |  | IE2 | 0.921 |
|  | Reflective engagement | RE1 | 0.776 |
|  |  | RE2 | 0.904 |
|  | Relational engagement | RLE1 | 0.803 |
|  |  | RLE2 | 0.918 |
| CII | Cultural identity integration | CII1 | 0.765 |
|  |  | CII2 | 0.793 |
|  |  | CII3 | 0.812 |
|  |  | CII4 | 0.828 |
|  |  | CII5 | 0.844 |
|  |  | CII6 | 0.861 |
| EP | English proficiency | EP1 | 0.781 |
|  |  | EP2 | 0.804 |
|  |  | EP3 | 0.826 |
|  |  | EP4 | 0.839 |
|  |  | EP5 | 0.852 |
| ICC | Cultural understanding | CU1 | 0.751 |
|  |  | CU2 | 0.823 |
|  |  | CU3 | 0.889 |
|  | Intercultural sensitivity | IS1 | 0.768 |
|  |  | IS2 | 0.846 |
|  |  | IS3 | 0.903 |
|  | Communication strategies | CS1 | 0.781 |
|  |  | CS2 | 0.852 |
|  |  | CS3 | 0.915 |
|  | Pragmatic appropriateness | PA1 | 0.774 |
|  |  | PA2 | 0.861 |
|  |  | PA3 | 0.922 |
|  | Multimodal cue interpretation | MCI1 | 0.806 |
|  |  | MCI2 | 0.932 |
|  | Interactional effectiveness | IEF1 | 0.789 |
|  |  | IEF2 | 0.876 |
|  |  | IEF3 | 0.917 |

# Supplementary File 4. Study 2 item-level standardized loadings

Manuscript: AI-Enhanced Social Media Affordances and Intercultural Communication Competence: Engagement, Identity Integration, and English Proficiency

This section reports standardized loadings for all 51 retained items from the Study 2 measurement model.

## Supplementary Table S4. Study 2 item-level standardized loadings

Note. Standardized loadings are based on the Study 2 measurement model.

| **Construct** | **Dimension** | **Item code** | **Standardized loading** |
| --- | --- | --- | --- |
| AIESMA | Algorithmic cultural exposure | ACE1 | 0.828 |
|  |  | ACE2 | 0.872 |
|  |  | ACE3 | 0.899 |
|  | AI-mediated comprehension support | MCS1 | 0.821 |
|  |  | MCS2 | 0.866 |
|  |  | MCS3 | 0.892 |
|  | AI-assisted expression support | AES1 | 0.84 |
|  |  | AES2 | 0.884 |
|  |  | AES3 | 0.911 |
|  | AI-enabled pragmatic feedback | APF1 | 0.836 |
|  |  | APF2 | 0.878 |
|  |  | APF3 | 0.906 |
|  | AI-supported cultural interpretation | SCI1 | 0.853 |
|  |  | SCI2 | 0.901 |
|  |  | SCI3 | 0.918 |
| IIE | Content engagement | CE1 | 0.914 |
|  |  | CE2 | 0.936 |
|  | Interactive engagement | IE1 | 0.921 |
|  |  | IE2 | 0.946 |
|  | Reflective engagement | RE1 | 0.918 |
|  |  | RE2 | 0.941 |
|  | Relational engagement | RLE1 | 0.916 |
|  |  | RLE2 | 0.938 |
| CII | Cultural identity integration | CII1 | 0.792 |
|  |  | CII2 | 0.811 |
|  |  | CII3 | 0.826 |
|  |  | CII4 | 0.839 |
|  |  | CII5 | 0.852 |
|  |  | CII6 | 0.866 |
| EP | English proficiency | EP1 | 0.814 |
|  |  | EP2 | 0.827 |
|  |  | EP3 | 0.842 |
|  |  | EP4 | 0.853 |
|  |  | EP5 | 0.861 |
| ICC | Cultural understanding | CU1 | 0.864 |
|  |  | CU2 | 0.901 |
|  |  | CU3 | 0.923 |
|  | Intercultural sensitivity | IS1 | 0.872 |
|  |  | IS2 | 0.904 |
|  |  | IS3 | 0.931 |
|  | Communication strategies | CS1 | 0.881 |
|  |  | CS2 | 0.912 |
|  |  | CS3 | 0.936 |
|  | Pragmatic appropriateness | PA1 | 0.886 |
|  |  | PA2 | 0.918 |
|  |  | PA3 | 0.943 |
|  | Multimodal cue interpretation | MCI1 | 0.889 |
|  |  | MCI2 | 0.941 |
|  | Interactional effectiveness | IEF1 | 0.875 |
|  |  | IEF2 | 0.908 |
|  |  | IEF3 | 0.934 |

**Supplementary File 5. Additional discriminant-validity summary**

Manuscript: AI-Enhanced Social Media Affordances and Intercultural Communication Competence: Engagement, Identity Integration, and English Proficiency

This section summarizes the HTMT and Fornell-Larcker evidence used to assess discriminant validity.

## Discriminant-validity evidence

Table S5 summarizes the construct-level discriminant-validity evidence.

## Supplementary Table S5. Additional discriminant-validity summary

| **Diagnostic** | **Result** | **Interpretation** |
| --- | --- | --- |
| HTMT | All values < .85; maximum = .714 | Supported discriminant validity |
| Fornell-Larcker | Applicable AVE square-root values exceeded corresponding construct correlations | Complementary evidence of discriminant validity |

Note. AIESMA was modeled as a reflective-formative higher-order construct; therefore, the conventional Fornell-Larcker diagonal is not directly applicable to the higher-order AIESMA construct.

## Supplementary File 6. Fornell-Larcker discriminant-validity evidence

Manuscript: AI-Enhanced Social Media Affordances and Intercultural Communication Competence: Engagement, Identity Integration, and English Proficiency

This file reports the Fornell-Larcker matrix supplementing the HTMT results in the main manuscript.

## Supplementary Table S6. Fornell-Larcker criterion and construct correlations

| Construct | AIESMA | IIE | CII | EP | ICC |
| --- | --- | --- | --- | --- | --- |
| AIESMA | N/A |  |  |  |  |
| IIE | 0.602 | 0.862 |  |  |  |
| CII | 0.322 | 0.437 | 0.831 |  |  |
| EP | 0.342 | 0.413 | 0.374 | 0.84 |  |
| ICC | 0.51 | 0.6 | 0.615 | 0.421 | 0.858 |

Note. Diagonal values represent the square root of AVE where applicable; off-diagonal values represent construct correlations. AIESMA was modeled as a reflective-formative higher-order construct, so N/A is retained where the Fornell-Larcker diagonal is not directly applicable. For IIE, CII, EP, and ICC, each applicable diagonal exceeded its construct correlations. This evidence complements the HTMT results in main-manuscript Table 7.

**Supplementary File 7. Common Method Variance Diagnostics**

Manuscript: AI-Enhanced Social Media Affordances and Intercultural Communication Competence: Engagement, Identity Integration, and English Proficiency

This file documents common method variance diagnostics reported in the main manuscript.

## Diagnostic results

| **Diagnostic** | **Estimate** | **Reference value** | **Interpretation** |
| --- | --- | --- | --- |
| Harman single-factor test | First factor = 31.42% of total variance | Below 50% | Did not indicate a dominant single-factor pattern |
| Full collinearity VIF | Maximum full collinearity VIF = 2.71 | Below 3.3 | Did not indicate a dominant full-collinearity pattern |

Note. These diagnostics did not indicate a dominant single-factor or full-collinearity pattern, but residual common method variance cannot be ruled out.

**Supplementary File 8. Robustness checks**

Manuscript: AI-Enhanced Social Media Affordances and Intercultural Communication Competence: Engagement, Identity Integration, and English Proficiency

This section summarizes robustness checks involving platform-exposure grouping and alternative construct specifications.

## Supplementary Table S8. Robustness-check summary

| **Robustness check** | **Specification** | **Result** | **Interpretation** |
| --- | --- | --- | --- |
| Platform-exposure grouping | Domestic-platform-dominant versus foreign-platform-exposed groups | Focal association directions and hypothesis decisions were substantively consistent across the domestic-platform-dominant and foreign-platform-exposed groups. | This sensitivity analysis was not intended as a formal test of measurement or structural invariance. |
| Alternative specifications of AIESMA or ICC | Disaggregated dimensions or alternative construct specifications | Alternative specifications of AIESMA or ICC produced substantively consistent structural conclusions. | The analysis was treated as a sensitivity check rather than confirmatory evidence. |

Note. These checks were treated as sensitivity analyses rather than confirmatory tests.

**Supplementary File 9. Alternative model comparisons**

Manuscript: AI-Enhanced Social Media Affordances and Intercultural Communication Competence: Engagement, Identity Integration, and English Proficiency

This section summarizes alternative model comparisons used to assess sensitivity to structural-model specification. The comparisons support statistical plausibility only and do not confirm temporal ordering.

## Supplementary Table S9. Alternative model comparison summary

| **Model** | **Specification change** | **Comparison** | **Interpretation** |
| --- | --- | --- | --- |
| Alternative Model 1 | Reversed IIE/CII ordering | The reversed IIE/CII ordering was examined as a structural sensitivity specification. The comparison did not provide evidence of temporal ordering; the proposed sequence remained theoretically specified rather than temporally established. | The comparison did not establish temporal ordering. |
| Alternative Model 2 | Removal of the nonsignificant direct AIESMA–ICC path | Removing the nonsignificant direct AIESMA–ICC path produced substantively similar conclusions regarding the indirect associations. | The substantive interpretation of the indirect associations was unchanged. |
| Alternative Model 3 | Alternative construct specifications | Alternative construct specifications produced substantively consistent conclusions. | This specification was treated as a sensitivity analysis rather than confirmatory evidence. |

Note. The comparisons were treated as sensitivity analyses and were not interpreted as evidence of model correctness.

**Supplementary File 10. Test-based relative English-proficiency robustness check**

Manuscript: AI-Enhanced Social Media Affordances and Intercultural Communication Competence: Engagement, Identity Integration, and English Proficiency

This section reports the supplementary moderation check based on valid reported scores from CET-4, CET-6, IELTS, TOEFL, or BEC. It represents relative standing within test type and does not imply absolute equivalence across tests.

## Supplementary Table S10. Test-based relative English-proficiency robustness check

| **Field** | **Detail** |
| --- | --- |
| Indicator set | CET-4, CET-6, IELTS, TOEFL, and BEC only |
| Score selection | Each respondent contributed one primary test indicator; when multiple valid scores were available, the score closest in time to the survey was selected. |
| Standardization | Scores were standardized within each test type using z = (individual score − mean for that test) / standard deviation for that test. |
| Sample coverage | N = 476 |
| Model role | Supplementary moderator; test type indicators were included as controls; not part of the main latent EP construct. |
| Key result | β = .087; SE = .039; t = 2.231; p = .026; 95% CI [.011, .163] |
| Interpretation | Directionally consistent with the main moderation result; represents relative standing within test type, not absolute equivalence across tests, and does not replace the main estimate. |

Note. This supplementary check does not replace the main self-reported EP moderation estimate. Test type indicators were included as controls.
